# Assessing effects of vibroacoustic stimulation compared to a guided mindfulness meditation using the biosignal of human speech

**DOI:** 10.3389/fnetp.2026.1677209

**Published:** 2026-03-13

**Authors:** Charlotte Fooks, Oliver Niebuhr

**Affiliations:** Centre for Industrial Electronics, University of Southern Denmark, Sønderborg, Denmark

**Keywords:** biosignal, speech prosody, stress, vibroacoustic, well-being, network physiology, mindfulness, meditation

## Abstract

**Introduction:**

High stress and low wellbeing pose severe individual, societal and economic threats, and there is a pressing demand for non-invasive stress reduction tools. This exploratory pilot study assessed the efficacy of speech prosody as a biosignal for stress elicitation, when comparing relaxation outcomes of two interventions with a control group.

**Method:**

Thirty participants were divided into three treatment groups; (1) guided mindfulness meditation (2) vibroacoustic intervention (3) control. All participants read aloud a text before and after one 20-min treatment. The sixty readings were assessed using a multi-parametric acoustic-prosodic analysis, and within-speaker differences were compared between the initial and final reading.

**Results:**

Results show groups (1) and (2) spoke with a breathier vocal quality in the second reading, while group (3) speech was tenser and at a lower, less variable loudness.

**Discussion:**

Results demonstrate speech prosody is a sensitive biomarker for treatment-effect classification and evaluation. Practical limitations and future research perspectives are discussed.

## 1 Introduction

High stress and low wellbeing across age groups continue to escalate globally, with stress posing severe societal and economic threats worldwide (World Health Organisation, 2023b; Council of the European Union, 2024). Concerns about the mental health and wellbeing of young people have gained significant public health attention, with growing evidence indicating that university students represent a particularly high-risk group for stress, psychological distress and mental health issues (Sommer, 2017; Bortes et al., 2021; Hammoudi Halat et al., 2023). Both physical and psychological aliments caused by stress are increasing world-wide, with 5% of adults suffering from depression (World Health Organisation, 2023a). In the EU, over half of all employees acknowledge widespread work-related stress, with 80% of managers experiencing its effects (End Stress EU, 2025). Beyond extensive economic impacts on industry and healthcare services, notably individuals experiencing chronic stress have reduced motivation, productivity, and creativity due to prolonged exposure to stress hormones (Putra et al., 2023) from interactions between nervous and endocrine systems. Physiological networks in the brain and body are in constant dialogue and determine psychological and physiological states. Novel stress recognition tools advance understanding and classification of physiological network interactions. This research investigates speech prosody as a non-invasive biosignal for stress classification, as representative of stress-response changes in the autonomic nervous system. The work assesses this by comparing two stress-reduction tools–a guided mindfulness meditation (Taren et al., 2015) and a vibroacoustic intervention (Fooks and Niebuhr, 2024a) – with a Control.

Speech prosody is the melody and rhythm of speech (Arvaniti, 2020). Prosody parameters modulate speech consciously and unconsciously and are accurate unbiased markers of physiological and psychological network states. As a highly sensitive biomarker, speech prosody can be used to non-invasively quantify physiological network changes. A benefit of using this biosignal to measure stress is that accurate data acquisition requires neither specific researcher knowledge nor action, and it can be collected with minimal participant instruction. For these reasons, it is less sensitive to both researcher and participant biases, compared to other stress-quantification metrics (Krumpal, 2013; Niebuhr and Neitsch, 2020). The current study assessed speech prosody as a stress marker in psychological and physiological networks, after exposure to either a vibroacoustic intervention or a guided mindfulness meditation. A specific set of prosodic parameters were used to analyse the effects of these Treatments on stress, as further discussed.

Research has consistently demonstrated that speech prosody is highly sensitive to mental stress, with measurable and systematic changes across multiple acoustic dimensions. Under increased cognitive load or psychological stress, speakers typically show elevated mean fundamental frequency (f0) of the voice. F0 is the main acoustic correlate of perceived voice pitch and, over time, a speaker’s intonation. Stressed speakers also show greater pitch variability in certain contexts, and altered intonation contours that reflect heightened arousal and emotional strain (Pakhomov and Kotlyar, 2011; Neubauer et al., 2017; Kappen et al., 2022b). A study by Kappen et al. shows robust patterns of intonation flattening and reduced pitch variation under cognitive load in ecologically valid tasks (Kappen et al., 2022a) that underscore systematic prosodic adaptation to mental demands. These changes have been observed both in controlled paradigms and in realistic, dyadic team settings, where prosodic shifts often co-occur with physiological stress markers, such as elevated heart rate and reduced heart rate variability (Neubauer et al., 2017).

Research has also shown speech prosody to be highly sensitive to physical stress and exertion, revealing consistent patterns of acoustic adaptation during activities such as cycling, stepping, running, or sustained physical tasks. Under physical load, speakers typically exhibit elevated fundamental frequency (f0), increased vocal intensity, and greater perceived vocal effort, reflecting both increased respiratory drive and heightened physiological arousal (Bone et al., 2017; Weston et al., 2023). Prosodic timing is similarly affected: articulation rate slows, and speakers have longer and more frequent pauses, in part to accommodate respiratory demands and reduced breath support during exertion (Trouvain and Truong, 2015). Voice quality may become less modal, i.e., tenser, with altered spectral features as airflow control changes under load (Bone et al., 2017). These prosodic adaptations differ in systematic ways from those observed in emotional or mental stress, underlining the importance of considering task-specific stress types when designing speech-based monitoring systems (Truong et al., 2015).

The brain and body process and respond to stressful stimuli through network interactions between psychological and physiological systems. It’s this pairwise interaction that determines the lived experience of stress. Stress is a coordinated interplay between cognitive processing and the nervous, endocrine, and immune systems. Widely assessed stress-related physiological changes include alterations in heart rate (BPM and HRV), cognitive processing (EEG), perspiration (GSR) and respiration (RR), though there are more subtle physiological markers that are also adept stress metrics. Speech prosody is one of these, as it can both accurately and subconsciously depict emotion and stressed states. Alternating network interactions of physiological systems derive changes in prosodic speech patterns. Thus, stress classification using speech analysis can accurately depict changes in underlaying physiological system interactions and their network dynamics. Physiological and cognitive stress activate the sympathetic nervous system, which induces hypertonicity (muscle stiffness) across the intricate musculature involved in speech production. Specifically, increased tension in laryngeal muscles can elevate fundamental frequency (f0) and introduce vocal strain or tremor, while constricted respiratory muscles may lead to shallower breathing and reduced phonation control. Hypertonicity in the articulatory muscles (jaw, tongue, lips) can compromise the precision and range of motion necessary for clear articulation. These stress-induced alterations in speech motor control manifest acoustically as changes in pitch, voice quality, speaking rate, and an increase in disfluencies. In the context of Automatic Speech Recognition (ASR) technologies, these deviations from normative speech patterns significantly degrade performance, leading to elevated Word Error Rates (WERs) in networked speech systems, which hinder the intended seamlessness of human-machine interaction.

On the other hand, the sensitive reaction of speech and voice patterns to stress makes them effective biosignals for detecting and estimating degrees of experienced stress. Numerous studies have demonstrated that machine learning models can achieve substantial accuracy in detecting stress from speech, typically reporting performance levels in the 70%–90% range. Early work using support vector machines and acoustic-prosodic feature sets—including pitch, energy, and timing cues—achieved classification accuracies of around 75%–85% in controlled lab-induced stress scenarios (Paul et al., 2015). More recent approaches leveraging deep learning architectures, such as convolutional and recurrent neural networks, have further improved stress detection accuracy to approximately 80%–90%, particularly when trained on diverse, real-world speech data (Baird et al., 2021). In mobile health contexts, studies have shown that integrating speech features with physiological signals can yield even higher accuracy and robustness for stress monitoring applications on smartphones (König et al., 2021). A 2024 paper by the authors of the present study (Fooks and Niebuhr, 2024a) found acoustic-prosodic parameters alone (those of speech prosody) could accurately predict before/after Treatment readings with 85.9% accuracy using an LDA (Wilks-λ = 0.565, χ^2^ [13] = 39.618, p < 0.001). Altogether, these results highlight the applicability and accuracy of speech as a biomarker of mental and/or physiological stress.

A study by Fooks and Niebuhr (Fooks and Niebuhr, 2024a) assessed the pertinence of speech prosody as a biosignal to capture intervention effects on stress and wellbeing. The results indicated that prosodic parameters provide a robust marker of both psychological wellbeing and physiological stress, with acoustic features related to pitch and timbre playing a central role in this assessment (Patel et al., 2011; Van Puyvelde et al., 2018). The observed links between prosodic shifts and physiological stress changes were largely consistent with the literature reviewed above. In the same study, the authors also evaluated the wellbeing effects of a 45-min vibroacoustic intervention, showing that this duration was sufficient to increase wellbeing and reduce stress.

Building on this previous work, the present study adopts a stepwise, exploratory approach to further refine the temporal characteristics of effective relaxation interventions. Rather than assuming a fixed optimal treatment duration, the broader research aim is to identify how long an intervention must last to elicit measurable changes in speech-based stress markers, and whether shorter, more practical exposure times may produce detectable effects. This requires balancing feasibility considerations (i.e., testing multiple durations across studies) with the need to meaningfully assess effectiveness and compare intervention types.

Within this framework, the present study evaluates whether stress decreases and relaxation increases after a shorter 20-min exposure. Changes in physiological stress, operationalised via prosodic speech parameters, are compared across three conditions: guided mindfulness meditation, vibroacoustic stimulation, and a no-stimuli control. The study thus contributes to the development of experimental protocols for stress elicitation by examining whether speech prosody parameters indicative of relaxation and reduced stress can still be observed after a reduced intervention duration.

It is hypothesised that the speech parameters of intonation, vocal effort, and loudness will reflect changes indicative of physiological and psychological relaxation and reduced stress following a 20-min exposure, compared to a control group. Specifically, psychological relaxation and reduced stress are expected to produce the opposite prosodic pattern to that typically observed under stressful conditions, as outlined in the Introduction. That is, if prosody under stress is characterised by higher pitch and greater pitch variability, a louder, tenser, and more effortful voice, then a more relaxed, stress-reduced speaking style (relative to baseline) should be characterised by: (1) a lower pitch level, (2) flatter intonation contours (i.e., higher minima and lower maxima), (3) reduced loudness, (4) reduced vocal effort, and (5) reduced vocal tenseness (i.e., breathier voice quality).

Section 2.2.3 outlines how these elements (1)–(5) of the hypothesis are represented by acoustic-prosodic measures.

## 2 Materials and methods

### 2.1 Materials

#### 2.1.1 Vibroacoustic technology

Vibroacoustic technology is a hardware combining audible sound with synchronised tactile vibration. Apparatus constitutes chairs, beds, tables and other devices. Terminology for the technology is diverse, and includes rhythmic sensory stimulation, physio-acoustic treatment or therapy, vibroacoustic therapy, and VA treatment. It’s uses are extensive and multifaceted, including and not limited to; to pain reduction (Campbell et al., 2019), improved self-awareness and self-esteem (Rüütel et al., 2004), heightened relaxation (Patrick, 1999), and depression symptom reduction (Braun Janzen et al., 2019). Often, specifically designed sound and/or music is created for the modality, with speakers built into the hardware to emit kinetic low-frequency sonic vibration. The technology can decrease physiological stress by reducing sympathetic nervous system activity (Delmastro et al., 2018; Kantor et al., 2022b).

#### 2.1.2 Meditation

Meditation is a cognitive practice supporting relaxation and stress reduction. It is a process of non-judgemental self-reflection that originated in the Eastern hemisphere and became popularised in the West in the mid-20th century by medical practitioners and alternative medicine advocates (Kabat-Zinn, 2018; Chopra, 2020). A guided mindfulness meditation was used in the present study which, through practitioner narration, prompted participants to focus cognisance on presence in the physical body. Calming ambient music featured as a background accompaniment to aid relaxation and stress reduction. Meditative focused attention is characterised by calm, positive emotional experiences, and a reduction in both psychological and physiological stress (Goyal et al., 2014; Sandler et al., 2016). Research indicates the efficacy of meditation to mediate the vagus nerve, which is a main component for parasympathetic nervous system regulation. Vagal tone regulates the stress response and is increased with meditation. Meditation affects vagal tone by increasing parasympathetic nervous system activity, causing a physiological relaxation response (Breit et al., 2018).

### 2.2 Methods

#### 2.2.1 Procedure

Thirty participants between the ages of 27–50 (9 males/21 females) voluntarily took part in the study. Being a pilot study for the reasons put forward in the Introduction, the sample size was chosen to balance practical feasibility with the need to obtain initial estimates of effect patterns and variability.

The research adopted a before-after-treatment experimental design and constituted three stages: a before-treatment reading (BEF), the relaxation treatment (RELX-), and an after-treatment reading (AFT). For both BEF and AFT conditions, participants read the same text aloud; BEF served as a control baseline for speech prosody, to which AFT was compared. Participants experienced one Treatment only (see Table 1). For the RELX- condition (Treatment), participants were split into one of three Treatment groups: a vibroacoustic intervention (RELX-VBRO), a guided mindfulness meditation (RELX-MEDI), or a no-stimuli control group (RELX-CONT). This three-level factor is referred to as Treatment.

**TABLE 1:** Flow diagram of the study stages: each participant experienced one Treatment (RELX-).

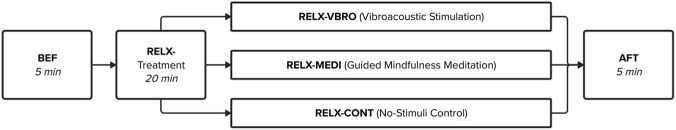

Participants were randomly assigned to the three Treatment conditions (RELX-VBRO, RELX-MEDI, RELX-CONT) using a stratified block randomisation procedure based on gender. Given the well-documented gender-related differences in several acoustic voice parameters, gender was considered the only relevant balancing factor. Separate randomisation lists were created for female and male participants, and participants were allocated in blocks to ensure an equal gender distribution across conditions. This resulted in balanced group compositions of seven female and three male participants per condition. No additional stratification variables were used, as no other participant characteristics are (at least at this stage) known from prior research to systematically influence within-speaker prosodic change in this type of data collection. For example, the data obtained by Fooks and Niebuhr (2024b) suggest no age differences in how RELX-VBRO treatment affects participants (physiological) responses.

The study adopted a within-subjects experimental design with all 30 participants experiencing all three stages (BEF, RELX-, AFT). Both the BEF and AFT conditions were allocated 5 min in the study design and took approximately 2 min to complete. All participants experienced one relaxation treatment (RELX-), which lasted for 20 min. The authors compared the stress and wellbeing effects of the RELX- conditions. For each participant a total session duration was 40 min as it included a short pre-study briefing and post-debriefing. The experiment was conducted at the National Institute of Public Health in Copenhagen, Denmark, and participants were assessed individually. The experimental setting was designed with soft lighting to reduce effects of a clinical study environment on result outcomes. A sight-blocking wall panel was placed between the researcher and participant throughout to reduce priming and facilitate participant comfort.

Two stress-reducing relaxation interventions were compared in the conditions RELX-VBRO (vibroacoustic intervention) and RELX-MEDI (guided mindfulness meditation). RELX-CONT was a no-stimuli control Treatment that served as a control baseline for the relaxation interventions. All participants experienced one RELX- condition. For the 20 min duration of all three RELX- conditions participants: lay in the supine position on top of a vibroacoustic module (turned off when not in use); had closed eyes; wore Bose QuietComfort 35 II noise cancelling headphones. The vibroacoustic module used for the RELX-VBRO condition was a “The Viboard 3.0” of the Danish startup vibroacoustics.dk (VibroAcoustics, 2024). It is a wooden box the size of a single mattress with two tactile transducer speakers evenly spaced on the underside of the board that enable audible-tactile sound.

For the RELX-VBRO condition, a specifically designed soundscape was used in the study which can be retrieved upon request to the first author. It included live recordings of natural sounds (birdsong, insects and running water (Buxton et al., 2021)) which were used intentionally and consistently throughout. Instruments featured were those typically used in sound baths (Parker, 2018) including a kenari seed nutshell shaker, tuning forks, a matsu bell shaker and a muted conga drum. Electronic instrumentation included a 1980s shoegaze-style keyboard, of which individual notes were played simplistically rather than as chord progressions, in addition to a periodical ‘whoosh’ sound effect with a short attack and long decay and sustain. An electronic didgeridoo that included pitch sequences and solfeggio frequencies postulated to have positive physiological and psychological properties was also present (Calamassi and Pomponi, 2020; Yang et al., 2023). The track was unscored by a pulsed 40 Hz frequency achieved by amplitude modulation. 40 Hz was used as it is consistently shown to be most efficacious in vibroacoustic studies (Bartel et al., 2017). Postulations for the aptitude of this frequency in vibroacoustic contexts include: transducer efficiency increases in the lower frequency range; vibrotactile sound needs to be more than 27 Hz for physical perceptibility; and 40 Hz is the most evocative frequency for brainwave entrainment to occur, as it is the frequency range at which the gamma band oscillates (Fries, 2009; Kantor et al., 2022a). Gamma brainwaves have the farthest-reaching cranial range–attaining the ability to facilitate connections between all hemispheres and lobes–thus they are associated with many cortical processes including emotion, arousal, concentration, and sensory integration (Jirakittayakorn and Wongsawat, 2017).

For the RELX-MEDI condition, a guided mindfulness meditation narrated by wellbeing practitioner Shamash Alidina was used (Alidina, 2024). Ambient background music in the recording that accompanied the meditation practice constituted electronic string harmonics between 40 Hz and 550 Hz. This harmonic 16-bars of string instrumentation was looped in the recording and delimited by a periodic triangle that sounded after each repetition. Layered on top of this, narrated meditative prompts required participants to mentally scan their body starting with the toes and ending in the head, while projecting self-compassion and gratitude toward various aspects of themselves and their life (including in their relationships, able-bodiedness, career etc.). This recording can also be assessed upon request to the corresponding author. Both vibroacoustic technology and guided mindfulness meditation have been shown to increase physiological and psychological relaxation and reduce stress (Goyal et al., 2014; Bartel et al., 2017; Valosek et al., 2021; Kantor et al., 2022b; Vilímek et al., 2022).

#### 2.2.2 Elicited text

For the BEF and AFT conditions, participants read the same short text aloud. “The Rainbow Passage” is a phonetically balanced English-language text that is widely used in speech research. Compared to other speech science texts, this was chosen as it elicits emotionally coloured speech, with 17.5% high-arousal words and 5.5% less-familiar terms (Ben-David et al., 2016). Throughout both the BEF and AFT conditions, participants sat behind a free-standing acoustic panel to ensure the text was naturally read. To avoid participant biases, no instructions were given other than to read the text aloud. Recordings were made at 48 kHz sampling rate and 24-bit quantization. A highly directional (super-cardioid) Zoom SSH-6 shotgun microphone was used to record the speech. It was pointed at the participants mouth and obscured from view. All best-practice guidelines as summarised in Niebuhr and Michaud (2015) (Niebuhr and Michaud, 2015) were taken into account regarding room acoustics, familiarisation phases, and participant instruction.

#### 2.2.3 Speech prosody analysis

The maximum amplitude of all audio recorded during the BEF and AFT conditions was normalised to 99% of the available dynamic range of the signal prior to analysis. Recordings were post-processed with a script (de Jong and Wempe, 2009) written for PRAAT (Seong, 2022) that marked and separated intermittent pauses from speech sections. The number of silent pauses and their average duration was determined per participant. In addition, a Python script of the second author was used to conduct an acoustic-prosodic analysis. Prosodic features measured in the speech recordings were the fundamental-frequency (f0) minimum, maximum, range, variability (standard deviation) and level (in Hz) as well as the total number and mean duration of silent pauses. In addition, the speaking rate was determined in syllables per second, and the loudness level and variability of the produced speech in terms of RMS (dB SPL) of the signal elongations (based on a 20 ms time window). To estimate vocal effort and timbre (or voice quality), the lower formant frequency levels F1, F2 and F3 (in Hz) and the levels and variabilities of the spectral slope estimates H1-H2, H1-A1, H1-A2 and H1-A3 were measured.

The formant frequencies (F1, F2, F3) and acoustic-prosodic spectral-tilt estimates (H1-H2, H1-A1, H1-A2, H1-A3) are highly sensitive to speaker vocal effort and underlying voice quality. With increased vocal effort, speakers typically increase subglottal pressure and adjust vocal tract configurations—such as wider mouth opening and potential pharyngeal constriction—leading to an upward shift in F1 and often higher formants, contributing to a perception of increased loudness and a “shouty” or “pressed” voice quality (Traunmüller and Eriksson, 2000). Concurrently, spectral-tilt parameters provide crucial insights into the glottal source characteristics and their interaction with the vocal tract, thereby indicating voice quality. H1-H2 measure the relative amplitude of the first two harmonics: higher values signify a breathier voice, while lower or negative values indicate a more pressed or tense phonation. Similarly, H1-A1, H1-A2, and H1-A3 quantify the amplitude difference between the first harmonic and the corresponding formant peaks, reflecting the efficiency of glottal excitation at different vocal tract resonances. Increased vocal effort typically results in a less steep spectral tilt (i.e., reduced H1-H2, H1-A1, H1-A2, H1-A3), signifying a stronger glottal source with more energy in higher harmonics, characteristic of louder and more effortful speech (Klatt and Klatt, 1990; Hillenbrand et al., 1994). Together, these measures offer a comprehensive acoustic profile of how vocal production adapts to varying effort levels and manifests distinct voice qualities.

No gender-based normalisation was applied to the acoustic parameters. While systematic gender-related differences are well established for some measures (e.g., fundamental frequency and formant structure, see (Van Puyvelde et al., 2018), appropriate reference values and, thus, applicable correction factors are not consistently available for the full set of parameters analysed here—particularly for intensity-related measures and spectral-tilt indices as well as for our speakers native language background. Applying gender-based normalisation under such uncertainty risks introducing additional and potentially stronger systematic bias, rather than reducing the existing one.

In addition, the present analyses focus on within-speaker change between the before- (BEF) and after-treatment (AFT) conditions rather than on absolute between-group differences. This within-subject design, in combination with stratified block randomization (see. 3.2.1.), substantially reduces and balances the influence of inter-speaker variability, including stable gender-related baseline differences. Moreover, exploratory analyses revealed no indication of a systematic interaction between gender and Treatment condition. For instance, the qualitative pattern of prosodic changes remained comparable when analyses were restricted to female participants only, albeit with reduced statistical power due to the smaller subsample. Nevertheless, as we will also point out in the Discussion section, future studies with larger samples should explicitly test gender as a potential moderating factor and evaluate empirically grounded normalisation strategies where appropriate.

## 3 Results

### 3.1 Statistical model and general outcome

To analyse the results, within-speaker difference values were compared between the initial (BEF) and final (AFT) reading tasks. In other words, the reading tasks provided information about whether the relaxation intervention between the two tasks (RELX-VBRO, RELX-MEDI, or RELX-CONT) influenced participants’ voice acoustics in the final reading task (AFT) in a systematic manner, that is, in a comparable direction across speakers relative to their own baseline (BEF). This within-speaker approach was chosen to minimise the impact of inter-individual variability and to focus on treatment-related change rather than on absolute between-speaker differences.

A one-way multivariate analysis of variance (MANOVA) was conducted using Treatment (VBRO vs. MEDI vs. CONT) as a three-level fixed factor. Given the study design with only two measurement time points per participant and a relatively small sample size, more complex modelling approaches (e.g., linear mixed-effects models) were not expected to provide more robust or stable estimates and could risk overparameterisation. The present analytical strategy therefore prioritises transparency and robustness in line with the exploratory character of the study.

Due to the limited sample size and the pilot nature of the investigation, the significance threshold for alpha-error levels was set to p ≤ 0.1. This more liberal threshold was chosen to reduce the risk of Type II errors and to allow potentially meaningful effects to be identified in this early-stage investigation. Effect sizes are therefore reported alongside p-values to support a more nuanced interpretation of the findings. The authors acknowledge that this analytical decision prioritises sensitivity over strict statistical conservatism and should be taken into account when interpreting the results.

The results of the MANOVA show BEF to AFT differences in the speakers’ reading voices as a function of Treatment in three vocal dimensions: intonation (F [2,30] = 4.77, p = 0.02, ηp^2^ = 0.24), vocal effort level (F [2,30] = 2.33, p = 0.1, ηp ^2^ = 0.13), and loudness level and variation (F [2,30] = 3.25, p = 0.05, ηp ^2^ = 0.18; F [2,30] = 3.10, p = 0.06, ηp ^2^ = 0.17). These three effect domains form the structure of the following Results sections. Thus, in the remainder of this section, intonation, vocal effort/voice quality, and loudness are addressed in turn, with reference to the individual acoustic parameters.

Furthermore, for clarity, all figures in the following subsections visualise within-speaker difference values (BEF–AFT), not the absolute values of the two reading tasks. Positive mean values therefore reflect lower average values in the AFT condition relative to BEF, whereas negative mean values indicate that AFT values were on average higher than in the BEF condition.

### 3.2 Results on intonation

**FIGURE 1:**
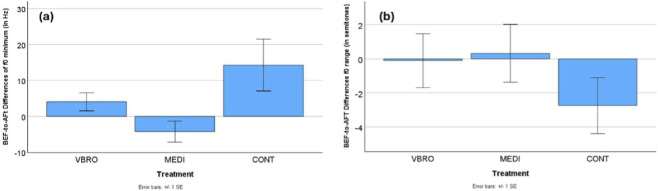
Mean within-speaker difference values (BEF–AFT) and standard error bars for **(a)** f0 minimum and **(b)** f0 range across the three treatment conditions (VBRO, MEDI, CONT; N = 30). Positive values indicate lower average values in the AFT condition compared to BEF, whereas negative values indicate higher values in AFT than in BEF.

The intonation dimension created the strongest effect in the data. As is shown in Figure 1a, the BEF-to-AFT differences in the speaker’s f0 minimum resulted in clearly larger values in the RELX-CONT than in the other two conditions of RELX-VBRO and RELX-MEDI. A larger difference value means that the f0 minimum in the BEF readings was higher than in the AFT readings. Compared to the control-group (CONT) change, the BEF-to-AFT differences in the RELX-VBRO and RELX-MEDI conditions were close to zero or slightly below zero, pointing to f0 minimum being almost at the same level in AFT as in BEF (p < 0.05 according to multiple-comparisons t-tests). There was no significant difference between VBRO and MEDI in terms of the f0 minimum differences from BEF to AFT Treatment, i.e., both VBRO and MEDI affected the speakers’ f0 minimum productions equally. Figure 1b shows that the f0 range was generally larger in the AFT condition for the CONT group, compared to BEF (p < 0.05). In contrast, it slightly shrank in the other two Treatment conditions VBRO and MEDI–though this range effect of Treatment did not reach statistical significance (p = 0.20).

### 3.3 Results on vocal effort

**FIGURE 2:**
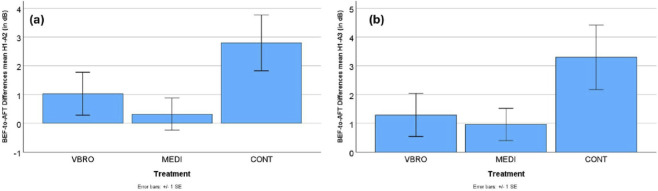
Mean within-speaker difference values (BEF–AFT) and standard error bars for **(a)** H1-A2 and **(b)** H1-A3 across the three treatment conditions (VBRO, MEDI, CONT; N = 30). Positive values indicate lower average values in the AFT condition compared to BEF, whereas negative values indicate higher values in AFT than in BEF.

For the prosodic dimension of vocal effort, the BEF-to-AFT difference values of the spectral-tilt estimates were found to be consistently larger for the CONT group speakers than for the VBRO and MEDI speakers (all p = 0.05 or p < 0.1 according to multiple comparisons t-tests). Larger difference values here mean the spectral tilt became smaller after treatment (AFT), indicating increased vocal tenseness/effort. Since vocal tenseness or effort is negatively linked to spectral tilt (with smaller or negative values indicating a tenser voice), this result means that the CONT group speaker voices were tenser and more effortful in the AFT than the BEF readings. Like for the intonation dimension, the MEDI and VBRO groups differed equally in that respect from the CONT group. Both VBRO and MEDI resisted this increase in vocal effort, showing no significant difference from each other with respect to vocal tenseness or effort. Figures 2a,b illustrate this results pattern for the H1-A2 and H1-A3 measures.

### 3.4 Results on loudness

**FIGURE 3:**
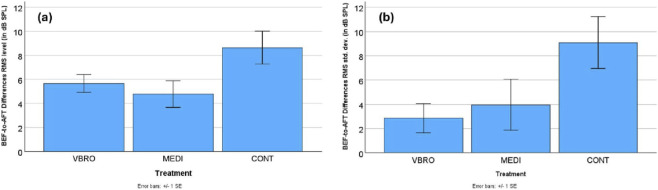
Mean within-speaker difference values (BEF–AFT) and standard error bars for **(a)** RMS level and **(b)** RMS variability (standard deviation) across the three treatment conditions (VBRO, MEDI, CONT; N = 30). Positive values indicate lower average values in the AFT condition compared to BEF, whereas negative values indicate higher values in AFT than in BEF.

With regards to loudness, results showed that when compared to the CONT group’s AFT readings, the AFT readings of the VBRO and MEDI groups showed smaller changes, i.e., difference values closer to zero. More specifically, the CONT group speaker readings got about 4–5 dB softer (in terms of RMS dB SPL) from BEF to AFT Treatment. In contrast, the VBRO and MEDI voices stayed significantly louder after the treatment (in terms of RMS dB SPL, p < 0.05). In addition, they remained more variable. That is, the VBRO and MEDI groups’ voices remained not just louder, but also more variable (i.e., their loudness fluctuated more). For all groups, variability got smaller after treatment (i.e., voices became more consistent in loudness). That is, the RMS standard deviation (in dB SPL) shrank from BEF to AFT readings for all three groups (i.e., there are positive difference values). This shrinking effect occurred more significantly for the CONT group speakers than for the VBRO and MEDI speakers (p < 0.05). Again, there were no significant differences between the two test conditions VBRO and MEDI. This results pattern is displayed in Figures 3a,b.

## 4 Discussion

In summary, this phonetic analysis reveals that the most pronounced and distinct prosodic changes were found for the speakers in the control group (CONT). Speaker pitch or intonation (measured by f0 in Hz) in the control group went down a lot in the AFT condition, with marginal effects seen for the other Treatments, as in Figure 1a. This indicates that the control group speakers (CONT) in the AFT condition spoke with a deeper voice. The range or fluctuation in speaker pitch (measured by f0 range in semitones) increased a lot for the control group in the AFT condition, with near-zero effects for both the vibroacoustic stimulation (VBRO) and guided mindfulness meditation (MEDI) conditions, see Figure 1b. This implies that the control group (CONT) speakers used a broader pitch range in the AFT condition. With respect to voice tenseness (measured by H1-A2) and vocal effort (measured by H1-A3), results showed that while vibroacoustic and meditation AFT speech was breathier, for the control group, speech was more tense and constricted, see Figures 2a,b. For speech loudness (measured with RMS level), results show that the control group spoke more quietly in the AFT condition, while the vibroacoustic and meditation groups remained significantly loud, as in Figure 3a. The loudness variability (calculated by RMS standard deviation) for all three Treatments shrank in the AFT condition–though it shrank to a greater degree for the control group than for the others. This means that variability in speaker loudness for the vibroacoustic and meditation groups was greater in the AFT condition than for the control group, as shown in Figure 3b.

The study hypothesis cannot be accepted in full and is only partially supported by the present data. The original hypothesis predicted five prosodic outcomes following the relaxation interventions: (1) lower pitch level, (2) flatter intonation contours, (3) reduced loudness, (4) reduced vocal effort, and (5) reduced vocal tenseness (i.e., breathier voice quality).

Of these five predicted outcomes, only changes in voice quality showed consistent support in the expected direction. Specifically, both the vibroacoustic and meditation groups exhibited a breathier vocal quality in the after-treatment readings compared to baseline, reflected in the spectral-tilt measures. In contrast, pitch-related parameters changed only marginally, loudness patterns partly shifted in the opposite direction, and several effects were weak or inconsistent across measures. These findings therefore suggest that a 20-min exposure to the two interventions is insufficient to induce a broad and systematic prosodic profile of relaxation as initially hypothesised, but may selectively affect aspects of voice quality.

The following interpretation should therefore be understood as an exploratory attempt to account for this selective pattern of results rather than as a confirmation of the original theoretical predictions.

In contrast to the two relaxation conditions, the most pronounced and consistent prosodic changes were observed in the control group. Control participants showed a marked decrease in pitch level (lower f0 minimum), an increase in pitch variability, reduced loudness, and a more tense or pressed voice quality in the after-treatment readings. This paradoxical co-occurrence of reduced melodic output with increased indicators of vocal effort may be informative and warrants closer examination, as increases in vocal effort are typically associated with greater loudness and elevated pitch levels (Traunmüller and Eriksson, 2000; Jessen et al., 2005). In the after-treatment readings for the control group, participants spoke with a more tense or pressed vocal quality which was concurrently quieter and less variable. Two possible interpretations may account for these outcomes: emotional suppression of psychological/cognitive stress, and/or mental fatigue, as further discussed.

It is postulated that the control group results may be due to a conflict between physiological and psychological states: while deeper, softer, quieter speech reflects a calm and rested physiological state—as participants lay down with their eyes closed for 20 minutes—the observed tense vocal effort might be explained by (a) cognitive stress arising from anticipating an active research task and then having to remain inactive for 20 min, and/or (b) fatigue or lethargy associated with reading a text aloud after a period of respite. Regarding (a), i.e., the suppression of psychological/cognitive stress, the activation of the sympathetic nervous system often leads to increased, involuntary, muscle tension throughout the laryngeal and articulatory systems. This emotion-driven laryngeal hyper-adduction (also referred to as hyperfunctional pattern) is a well documented phenomenon in logopedics and causes the vocal folds to close more tightly for longer periods (Seifert and Kollbrunner, 2005; Tomlinson and Archer, 2015; Van Puyvelde et al., 2018). Such prolonged closure affects the acoustic output of the voice, most notably by flattening the spectral tilt. Accordingly, spectral measures such as H1–H2 and H1–A3 decrease, which reflects a shift towards a more pressed or strained voice quality rather than a breathy or soft one. Note that this represents a qualitatively different type of “effort” than the well-established above cited vocal effort observed under loud speech, physical exertion, or large speaker-listener distances. Consequently, the otherwise robust coupling between increased vocal effort, flatter spectral tilt, and elevated f0 levels does not hold here. Instead, increased laryngeal constriction due to a hyperfunctional, stress-induced pattern co-occurs with reduced f0 variability and lower intensity, as observed in the present data.

Alternatively, the control group’s overall prosodic change could be due to a state of mental fatigue, coupled with a compensatory effort to maintain a good speech production performance to meet the experimental reading task requirements. While fatigue typically reduces vocal effort (Honig et al., 2014; Huckvale et al., 2020; Icht et al., 2020; Cañas et al., 2021), the increased effort may be a compensatory mechanism against vocal fatigue or insufficient respiratory support, but without other prosodic parameters joining in to create a vivid speech melody. In this instance, a low respiratory effort reduces the amount of air pressure driving the vocal folds, which causes the radiated acoustic signal’s root-mean-square amplitude to drop. More simply, the overall loudness of the sound is lower as less energy is used to generate the voice. Kohtz and Niebuhr (2013) (Kohtz and Niebuhr, 2013) and Niebuhr et al. (2018) (Niebuhr et al., 2018) report “prosodic erosion effects” related to monotonous or repeated reading of speaking tasks–the prosodic changes of which resemble those that were found here for the control group speakers (although not in all prosodic parameters). Given that the present study’s control group participants did nothing for 20 min, fatigue or lethargy could reasonably be expected.

Notably, both proposed mechanisms to explain the observed combination of increased vocal effort (flatter spectral tilt) with lower f0 values are explored here as theoretical interpretations rather than objective conclusions drawn from the data. Furthermore, it is important to note that, for almost all prosodic parameters, the direction of change from the BEF to the AFT reading was similar across the vibroacoustic, meditation, and control conditions. In other words, apart from the consistently breathier voice quality observed in the vibroacoustic and meditation groups, these two groups did not exhibit fundamentally different prosodic patterns compared to the control group. Instead, the key difference lay in the magnitude of change: prosodic shifts were significantly weaker following vibroacoustic stimulation and meditation than in the control condition.

Thus, the vibroacoustic and meditation treatments both ensured that the prosodic performance from the BEF reading task was retained to a far greater extent in the AFT reading task than for the control group. If this outcome is linked to the possible explanation(s) offered above, then the smaller extent of the prosodic changes from BEF to AFT readings in the VBRO and MEDI groups would indicate that these treatments had less of an effect on participant psychological/cognitive stress and fatigue. For example, the vibroacoustic and meditation group speakers may have experienced, in terms of their prosody, a higher level of wellbeing or stimulation in their AFT treatment reading than the control group speakers. Of course, this conclusion is speculative and exploratory, but there is evidence from previous studies (Fooks and Niebuhr, 2024b) using EEG data that the vibroacoustic treatment has less of a relaxing and soporific effect on users, and more of a stimulating and focusing effect. Participants experienced mental stimulation or arousal both during and after a vibroacoustic treatment, which aligns with first-person accounts reported in after-treatment interviews with the researcher.

The present study aimed to assess the efficacy of two relaxation modalities for stress reduction compared to a control condition. Although breathier vocal quality is typically associated with a relaxed physiological state, the observed effects were not sufficiently robust to provide clear evidence for wellbeing or stress-reduction outcomes based on speech prosody alone. At the same time, previous research has established both vibroacoustic stimulation and mindfulness meditation as effective wellbeing interventions. The present findings therefore suggest that a 20-min exposure may be insufficient to induce strong, stable, or reliably detectable changes in prosodic speech markers. From this perspective, the current study provides an empirically grounded calibration point, suggesting that subsequent studies should prioritise longer exposure times (e.g., around 30 min or more) to better capture differentiated treatment effects while maintaining ecological feasibility.

Several limitations should be considered when interpreting these results. The relatively small sample size (N = 30) limited statistical power; consequently, in line with the exploratory nature of the study, a significance threshold of p ≤ 0.1 was adopted to reduce the risk of overlooking potentially meaningful effects. While this approach is common in pilot research, the present findings should primarily be interpreted as hypothesis-generating and warrant cautious interpretation. Moreover, the proposed physiological mechanisms remain speculative and require empirical substantiation.

A further methodological limitation concerns the absence of gender-based normalisation. Although the within-speaker design reduces the impact of inter-speaker variability on the primary analyses, future studies with larger samples should explicitly examine potential gender-related moderation effects and evaluate empirically grounded normalisation strategies to further strengthen population-level generalisability.

Future research should therefore employ larger samples and combine prosodic analysis with complementary measures such as questionnaires, self-reports, interviews, and additional physiological signals (e.g., HRV, EEG) to enable triangulation and a more comprehensive assessment of stress and wellbeing effects.

In addition, future longitudinal studies could assess sustained long-term intervention effects, with repeated exposure and defined timepoint analysis (T0 – immediately after exposure, T1 – 1 week after exposure, T2 – 2 weeks after exposure). This robust methodological design would support analysis and quantification of immediate-term sustained intervention effects, together with longer-term exposure outcomes. Additional stimuli variations could be explored, such as meditation narrator vocal quality (speech speed, pause duration, gender etc.), and differing genres, or removal of, meditative background music. These studies could also compare other stress-reduction interventions to assess if it is exposure time, the modality, or a combination of these that determines result outcomes. Prospective stress-reduction interventions worthy of exploration include short sleep stints or ‘power naps’, alternative sonic stimuli (such as field recordings of natural sounds), and Autonomous Sensory Meridian Response (ASMR) recordings. Extended research into stress reduction tools will optimise exposure times and highlight interventions that more rapidly modulate stress. A scoping review could compare modalities that induce fast physiological stress-reduction, as well as expose characteristic overlap–with respect to both networked system interaction affects and subjective relaxation outcomes. Results could be collated into the design of novel stress-reducing interventions. Finally, the present study illustrates that for comprehensive result analysis, future studies should combine physiological and psychological quantification metrics with prosodic assessments to depict a more detailed network interaction analysis. Collated biosignal data of this sort would illustrate how stress affects the inter- and intra- coordination and communication of human networked systems, and lead to the further development of more sensitive stress recognition and classification biometrics.

## Data Availability

The raw data supporting the conclusions of this article will be made available by the authors, without undue reservation. Python Programming Language (RRID:SCR008394) and Praat (RRID:SCR016564) were used for this study.
